# Maternal Obesity and Rectovaginal Group B Streptococcus Colonization at Term

**DOI:** 10.1155/2015/586767

**Published:** 2015-08-02

**Authors:** Shelby M. Kleweis, Alison G. Cahill, Anthony O. Odibo, Methodius G. Tuuli

**Affiliations:** Department of Obstetrics and Gynecology, Washington University School of Medicine, Campus Box 8064, 4566 Scott Avenue, Saint Louis, MO 63110, USA

## Abstract

*Objective*. To test the hypothesis that maternal obesity is an independent risk factor for rectovaginal group B streptococcus (GBS) colonization at term. *Study Design*. Retrospective cohort study of consecutive women with singleton term pregnancies admitted in labor at Barnes-Jewish Hospital (2004–2008). Maternal BMI ≥ 30 Kg/m^2^ (obese) or <30 Kg/m^2^ (nonobese) defined the two comparison groups. The outcome of interest was GBS colonization from a positive culture. Baseline characteristics were compared using Student's *t*-test and Chi-squared or Fisher's exact test. The association between obesity and GBS colonization was assessed using univariable and multivariable analyses. *Results*. Of the 10,564 women eligible, 7,711 met inclusion criteria. The prevalence of GBS colonization in the entire cohort was relatively high (25.8%). Obese gravidas were significantly more likely to be colonized by GBS when compared with nonobese gravidas (28.4% versus 22.2%, *P* < 0.001). Obese gravidas were still 35% more likely than nonobese women to test positive for GBS after adjusting for race, parity, smoking, and diabetes (adjusted OR 1.35 [95% CI 1.21–1.50]). *Conclusion*. Maternal obesity is a significant risk factor for GBS colonization at term. Further research is needed to evaluate the impact of this finding on risk-based management strategies.

## 1. Introduction

Group B streptococcus (GBS) was first recognized as a virulent pathogen responsible for most cases of neonatal sepsis in 1964 [1]. Since then, GBS has been associated with neonatal meningitis, pneumonia, septic abortions, and chorioamnionitis. It has also been implicated in puerperal sepsis, endometritis, and urinary tract infections [2, 3]. Bergqvist et al. showed that the vagina was the most likely source of GBS to the neonate as the antibiotic sensitivities of the neonatal organisms were similar to maternal vaginal cultures [2].

Intrapartum chemoprophylaxis has been shown to decrease early-onset neonatal GBS disease [4, 5]. In 1996 the American College of Obstetricians and Gynecologists, the Centers for Disease Control and Prevention (CDC), and the American Academy of Pediatrics (AAP) made recommendations for intrapartum prophylaxis to prevent perinatal GBS disease based on risk factors [6–8]. The CDC published revised guidelines in 2002 recommending universal culture-based screening at 35–37 weeks with intrapartum chemoprophylaxis for those who test positive. Risk-based treatment was recommended if GBS colonization status is unknown during labor. The risk factors considered included a prior affected infant, prematurity, prolonged rupture of membranes, and history of GBS bacteriuria during the current pregnancy, all of which are associated with increased risk of neonatal GBS disease.

Other maternal factors have been suggested as risk factors for GBS colonization. A number of studies have linked obesity to GBS colonization in both pregnant and nonpregnant women [9–11]. Further, obesity has been linked to an increased risk of early-onset neonatal GBS disease [12]. Most of the prior studies were based on population-based data with intrinsic limitations and some did not adequately control for potential confounders [13]. With the increasing rates of obesity, establishing an association with GBS colonization may inform risk-based intrapartum prophylaxis for women with unknown GBS status in labor.

The objective of this study was to investigate the association between obesity and GBS colonization in a term cohort. We tested the hypothesis that maternal obesity is an independent risk factor for rectovaginal GBS colonization at term.

## 2. Methods

This was a retrospective cohort study of all consecutive women with singleton term pregnancies admitted in labor at Washington University in St. Louis Medical Center from 2004 to 2008. We obtained informed consent and the study was approved by the Washington University School of Medicine Human Research Protection Office.

Women with documented GBS culture results and body mass index (BMI) information were eligible. We excluded women without GBS and BMI information and women delivering preterm (<37 weeks). Extensive data were extracted from the medical record including maternal sociodemographic factors, obstetric and gynecologic history, medical and surgical history, antenatal history, and obstetric outcomes. Maternal BMI was calculated from the patients' weight and height on admission. Obesity was defined using WHO criteria as BMI ≥ 30 Kg/m^2^. Term gestations were defined as gestational age of at least 37 0/7 weeks. Pregnancies were dated by a woman's last menstrual period and confirmed with first or second trimester ultrasonography using standard criteria. The outcome of interest was GBS colonization, defined as positive culture from a rectovaginal swab. Those individuals were treated as per CDC guidelines.

Maternal BMI ≥ 30 Kg/m^2^ (obese) or <30 Kg/m^2^ (nonobese) defined the comparison groups. Baseline characteristics were compared using univariable analysis. Continuous variables were compared using Student's *t*-test and categorical variables were analyzed using the Chi-square or Fisher's exact test as appropriate. Normality of distribution of the continuous variables was verified using the Kolmogorov-Smirnov test. The association between obesity and GBS colonization was assessed using adjusted and unadjusted odds ratios with 95% confidence intervals. Adjusted odds ratios were obtained using multivariable logistic regression to control for confounders. Candidate variables for the logistic regression models were selected on the basis of biologic plausibility, risk factors that have been identified in the literature, and results of our univariable analysis. Backwards elimination was used to reduce the number of variables in each model. Differences between hierarchical explanatory models were assessed using the likelihood ratio test or Wald test. Only factors contributing significantly to the explanatory model were included in the final model. Model fit was assessed with the Hosmer-Lemeshow goodness-of-fit test [14].

We included all consecutive subjects meeting inclusion criteria; no a priori sample size estimation was performed. Statistical tests were all 2-tailed and *P* < 0.05 was considered significant. All statistical analyses were completed using STATA software package, version 11, Special Edition (College Station, TX).

## 3. Results

**Figure 1:**
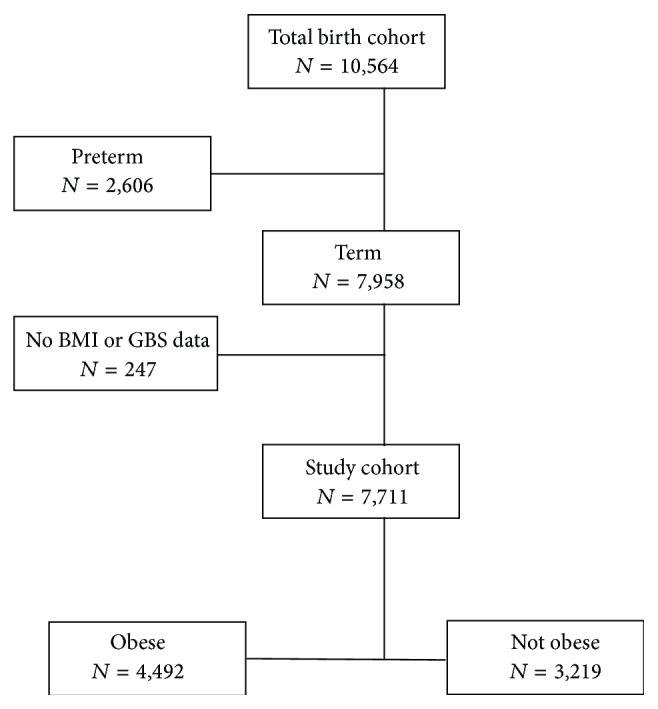
Flowchart of study subjects.

Of the 10,564 women admitted in labor at term during the study period, 7,711 met inclusion criteria (Figure 1). More than half of the cohort was obese (*n* = 4, 492, 58.3%). Majority of the women were African American (*n* = 5, 515, 71.5%) with a mean gestation age of 38.9 weeks. Obese and nonobese women were different in several baseline characteristics. Obese women were more likely to be older, Africa American, and having diabetes (Table 1).

The prevalence of GBS colonization in the entire cohort was relatively high (25.8%). Obese women were significantly more likely than nonobese women to be colonized by GBS (28.4% versus 22.2%, crude OR 1.39 (95% CI 1.25, 1.55), *P* < 0.001). After adjusting for race, parity, and diabetes, obese women were still 35% more likely to be colonized with GBS (adjusted OR 1.35 (95% CI 1.21–1.50)).

**Figure 2:**
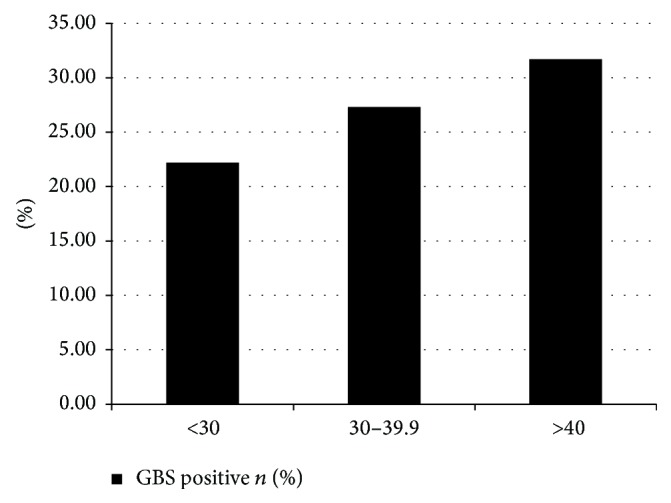
Risk of GBS colonization with increasing obesity categories.

To determine if there is a dose-response relationship between increasing BMI category and GBS colonization, we calculated the risk of GBS colonization for BMI < 30 Kg/m^2^, 30–39.9, and ≥40 Kg/m^2^. GBS colonization increased in a dose-response fashion with increasing BMI: 22.2%, 27.3%, and 31.7%, respectively (Figure 2). The trend persisted in multivariable analysis adjusting for confounders (Table 2).

## 4. Discussion

Widespread efforts, using a combination of screening and risk factor-based strategies, have resulted in a significant reduction in the incidence of early-onset neonatal sepsis due to GBS [15, 16]. Yet, GBS remains the leading cause of infectious mortality and morbidity in newborns [16]. While obesity has not been considered one of the risk factors for which intrapartum prophylaxis should be offered to women with unknown GBS status, our results show that obesity is associated with a 35% higher risk of GBS colonization at term.

The finding of an association between obesity and GBS colonization is consistent with prior studies [9–11]. Using population-based data, Stapleton et al. found a 20% increased risk of GBS colonization with obesity and 45% increase with severe obesity [11]. The 25.8% overall rate of GBS colonization in our cohort is relatively high but still falls within the 10–30% rates reported in the literature [17–20]. This higher rate may be attributable to a number of factors. First, our cohort is predominantly African American, a known risk factor for GBS colonization. Second, over 50% of our cohort was obese. Given the observed association between obesity and GBS colonization, this may explain the overall higher colonization rate. Third, prior studies report an increasing trend of GBS colonization over time. Stapleton et al. reported a progressive increase in GBS prevalence from 1997 to 2002, which was attributed to increased screening [11]. This may further explain the relatively higher rate noted in our contemporary cohort. Finally, our study differs from prior studies by including only women at term.

The biological mechanism for increased GBS colonization in obese women is unclear. However, it may be related to changes in the gastrointestinal microbial ecology with obesity. Animal and human studies demonstrate a shift towards increased Firmicutes (the phylum to which GBS belongs) and decreased Bacteroides with obesity [21]. These shifts reflect increased energy-reabsorbing potential of different ratios of Firmicutes and Bacteroides, especially in the digestion of fatty acids and dietary polysaccharides [22]. Further, a recent study showed that pregnancy itself is associated with changes in the gut microbiome similar to that seen in obesity [23].

Our study offers several strengths. This is a large analysis dedicated to evaluating the association between obesity and GBS colonization at term. The comprehensive delivery data compiled by trained research nurses enabled us to perform detailed analysis controlling for confounders. Further, by including only women at term, we focused on the subset of women in whom additional risk factors would influence clinical management if GBS status is unknown.

There are limitations that should be considered when interpreting our data. The retrospective nature of our study makes it vulnerable to selection bias, confounders, and inaccuracies in data collection. We included all consecutive patients meeting inclusion criteria, reducing the risk of selection bias. In addition, our database has been well validated by ongoing quality control, increasing confidence in our findings. While we controlled for confounders, there is the potential for residual confounding by variables we did take into account. Finally, our cohort is predominantly African American, raising the question of generalizability of our results. However, the persistent association after we controlled for confounders including race and the consistency of our results with other studies conducted among subjects of different demographics lend credence to the generalizability of our findings. Further studies in different populations will help further validate our findings.

One prior study found a higher risk of early-onset GBS sepsis in the neonates of obese women [12]. The results of that study coupled with our findings suggest that obesity may be both an important risk factor for GBS colonization and neonatal GBS disease. If these findings are confirmed by other studies, obesity may be considered in risk-based management strategies in women at term. Furthermore, a recent study reported a false negative antepartum culture rate of 9.8% [24], highlighting the potential role of risk factor based management strategies.

## 5. Conclusion

In conclusion, this large cohort study showed a significantly increased risk of GBS colonization in obese women at term. If confirmed by other studies, this finding, together with the reported higher risk of early-onset GBS in neonates of obese women, suggests that maternal obesity is a factor that needs to be considered in strategies for reducing GBS disease in neonates.

## Figures and Tables

**Table 1:** Characteristics of study subjects (*N* = 7,711).

Variable	Obese (*n* = 4492)	Nonobese (*n* = 3219)	*P*
Maternal age, mean (sd)	25.4 (5.9)	24.2 (6.2)	<0.001
Gestational age in weeks, mean (sd)	39.0 (1.2)	38.9 (1.2)	<0.001
Race, *n* (%)			
Black	3,380 (75.2)	2,135 (66.3)	<0.001
Caucasian	801 (17.8)	710 (22.1)	
Primiparous, *n* (%)	1,517 (33.8)	1,383 (43.0)	<0.001
Vaginal delivery, *n* (%)	2,875 (64.0)	2505 (77.8)	<0.001
Smoking, *n* (%)	794 (17.7)	594 (18.5)	0.381
Alcohol, *n* (%)	51 (1.1)	52 (1.6)	0.070
Illicit drug use, *n* (%)	414 (9.2)	356 (11.1)	0.008
Diabetes mellitus, *n* (%)	134 (3.0)	15 (0.47)	<0.001
Chronic hypertension, *n* (%)	212 (4.7)	37 (1.2)	<0.001

sd: standard deviation.

**Table 2:** Risk of GBS colonization with increasing obesity categories.

BMI category (Kg/m^2^)	GBS colonization *n* (%)	Unadjusted odds ratio (95% CI)	^*∗*^Adjusted odds ratio (95% CI)	*P* trend
<30 (*n* = 3,219)	713 (22.2)	Reference	Reference	<0.001
30–39.9 (*n* = 3,350)	914 (27.3)	1.31 (1.18, 1.48)	1.29 (1.15, 1.45)	
>40 (*n* = 1,142)	362 (31.7)	1.63 (1.40, 1.89)	1.54 (1.32, 1.79)	

^*∗*^Adjusted for race, parity, and diabetes.
